# The Role of Catechol-O-Methyl Transferase *Val(108/158)Met* Polymorphism (rs4680) in the Effect of Green Tea on Resting Energy Expenditure and Fat Oxidation: A Pilot Study

**DOI:** 10.1371/journal.pone.0106220

**Published:** 2014-09-19

**Authors:** Rick Hursel, Pilou L. H. R. Janssens, Freek G. Bouwman, Edwin C. Mariman, Margriet S. Westerterp-Plantenga

**Affiliations:** Department of Human Biology, Nutrition and Toxicology Research Institute Maastricht (NUTRIM), Maastricht University, Maastricht, The Netherlands; Pontificia Universidad Catolica de Chile, Chile

## Abstract

**Introduction:**

Green tea(GT) is able to increase energy expenditure(EE) and fat oxidation(FATox) via inhibition of catechol-O-methyl transferase(COMT) by catechins. However, this does not always appear unanimously because of large inter-individual variability. This may be explained by different alleles of the functional COMT *Val108/158Met* polymorphism that are associated with COMT enzyme activity; high-activity enzyme, COMT^H^(*Val/Val* genotype), and low-activity COMT^L^(*Met/Met* genotype).

**Methods:**

Fourteen Caucasian subjects (BMI: 22.2±2.3 kg/m^2^, age: 21.4±2.2 years) of whom 7 with the COMT^H^-genotype and 7 with the COMT^L^-genotype were included in a randomized, cross-over study in which EE and substrate oxidation were measured with a ventilated-hood system after decaffeinated GT and placebo(PL) consumption.

**Results:**

At baseline, EE, RQ, FATox and carbohydrate oxidation(CHOox) did not differ between groups. Significant interactions were observed between COMT genotypes and treatment for RQ, FATox and CHOox (p<0.05). After GT vs. PL, EE(GT: 62.2 vs. PL: 35.4 kJ.3.5 hrs; p<0.01), RQ(GT: 0.80 vs. PL: 0.83; p<0.01), FATox(GT: 18.3 vs. PL: 15.3 g/d; p<0.001) and CHOox(GT: 18.5 vs. PL: 24.3 g/d; p<0.001) were significantly different for subjects carrying the COMT^H^ genotype, but not for subjects carrying the COMT^L^ genotype (EE, GT: 60.3 vs. PL: 51.7 kJ.3.5 hrs; NS), (RQ, GT: 0.81 vs. PL: 0.81; NS), (FATox, GT: 17.3 vs. PL: 17.0 g/d; NS), (CHOox, GT: 22.1 vs. PL: 21.4 g/d; NS).

**Conclusion:**

Subjects carrying the COMT^H^ genotype increased energy expenditure and fat-oxidation upon ingestion of green tea catechins vs, placebo, whereas COMT^L^ genotype carriers reacted similarly to GT and PL ingestion. The differences in responses were due to the different responses on PL ingestion, but similar responses to GT ingestion, pointing to different mechanisms. The different alleles of the functional COMT *Val108/158Met* polymorphism appear to play a role in the inter-individual variability for EE and FATox after GT treatment.

**Trial Registration:**

Nederlands Trial register NTR1918

## Introduction

Overweight and obesity represent a rapidly growing threat to the health of populations worldwide and is caused by an imbalance between energy intake and energy expenditure (EE) [1], [2]. A negative energy balance is needed to produce weight loss and can be achieved by either decreasing intake or increasing expenditure [3], [4]. Amongst others, stimulation of EE by green tea (GT), rich in catechins and caffeine has attracted interest, especially because GT does not contain any energy itself, yet stimulates EE. Tea is made from the leaves of Camellia sinensis L. species of the Theaceae family, GT being the non-oxidized, non-fermented product, containing high quantities of several polyphenolic components such as epicatechin, epicatechin gallate, epigallocatechin and epigallocatechin gallate [5]. Caffeine has also been shown to stimulate thermogenesis and fat oxidation in humans [6]–[8]. The methylation of catechins by catechol-O-methyltransferase (COMT) and the inhibition of phosphodiesterase by caffeine appear to be the principal mechanisms behind the stimulating properties of GT. The importance of GT catechins in stimulating EE was shown by Dulloo et al. [9] who observed that the thermogenic effect of GT extract containing caffeine and catechins, is greater than that of an equivalent amount of caffeine. However, a thermogenic effect as well as a weight reducing effect has not been unanimously shown, indicating the presence of moderating factors. The difference in outcome between several ethnic populations suggests a role for genetic predisposition, which was supported by recent meta-analyses [10], [11] that addressed effects of GT on weight loss and thermogenesis. Different polymorphisms for COMT enzyme activity exist and these may be responsible for the variability in flavonoid O-methylation that was previously reported by Hodgson et al. [12]. The *Val(108/158)Met* polymorphism replaces valine by methionine, thereby changing enzyme activity. The inter-individual variability of the activity of COMT could vary as much as 3-fold. Moreover, there is evidence that there is a difference in COMT enzyme activity between ethnic groups [13]. Asian populations appear to have a higher frequency of the thermostable, high activity enzyme, COMT^H^ (*Val/Val* genotype) than the Caucasian populations that have a higher frequency of the thermolabile, low activity enzyme COMT^L^ (*Met/Met* genotype); half of Caucasians are homozygous for COMT^L^ (25%) or COMT^H^ (25%). The other 50% of this population is heterozygous (*Val/Met* genotype) [13]. This may explain the difference in sensitivity to interventions with GT, and why, in some studies with Caucasian subjects, no effect was seen after ingestion of GT. Hence, the aim of this pilot study was to examine the role of genetic predisposition in the effect of GT by measuring treatment induced EE and substrate oxidation after GT and placebo (PL) consumption in Caucasian subjects carrying either a COMT^H^ genotype or COMT^L^ genotype.

## Subjects and Methods

### Subjects

Fourteen healthy Caucasian subjects participated in this study after recruitment by advertisements in local newspapers and on notice boards at the university. All volunteers (N = 24) participated in an initial screening that involved measurements of body weight and height, blood sampling and included the completion of a questionnaire related to eating behavior (Three Factor Eating Questionnaire, TFEQ [14]) and the completion of a questionnaire related to health, use of medication, physical activity, alcohol consumption, food allergies, smoking behavior and daily caffeine consumption. All subjects were in good health, non-smokers, not using medication (except for contraception), at most moderate alcohol consumers and unrestrained eaters (as assessed by factor 1 of the TFEQ). Subject recruitment started in September 2009 and the study was conducted between February 2010 and June 2010. This study was conducted according to the guidelines of the Declaration of Helsinki and all procedures involving human subjects were approved by the Medical Ethical Committee of Maastricht University Medical Centre. Written informed consent was obtained from all subjects. The study was registered as follows: Nederlands Trial Register, registration number NTR1918. The protocol for this trial and supporting CONSORT checklist are available as supporting information; see Checklist S1 and Protocol S1.

### Experimental design

The study had a randomized, two arms, single-blind, crossover design. Subjects attended the university-laboratory once a week, during two consecutive weeks. They were instructed to abstain from caffeine-rich products like tea, coffee, cola-type soft drinks and energy drinks for at least 3 days before the test day. They traveled by public transport or by car, in order to avoid physical activity that would have increased EE at rest. Subjects arrived in the fasted state at 08.15h and were kept in a time-blinded surrounding. They emptied their bladder before the test. During the test subjects were lying in the supine position. After resting on a bed for 30 minutes, the EE at rest and the substrate oxidation of the subjects was measured for 30 minutes by means of an open-circuit, ventilated-hood system. Gas analysis was performed by a paramagnetic oxygen analyzer (omnical type 1155B, Crowborough Sussex, UK) and an infrared carbon dioxide analyzer (omnical type 1520/1507). Energy expenditure was calculated using Weir’s formula [15]. The RQ was calculated as CO_2_ produced/O_2_ consumed. Fat oxidation (FATox) and carbohydrate oxidation (CHOox) were calculated in grams/3.5 hrs with the formulas of Carpenter [16].

To test the effect of GT *vs*. PL on thermogenesis, subjects ingested in random order three GT capsules (Content: Sunphenon 90 LB, Taiyo Kagaku Co. Ltd, Mie, Japan; Capsules: Gelkaps, Falkenhagen, Germany), or the control, which were three PL capsules (Gelkaps, Falkenhagen, Germany), after measuring EE at baseline. In addition, subjects received water (100 ml) in order to swallow the capsules easily. The capsules all had the same appearance. The composition and the dose of the capsules are presented in **Table 1**. During the consumption of the capsules the hood was removed temporarily. After the ingestion the hood was placed back and the measurements continued for another 3.5 hours, during which the increase in thermogenesis was determined. Subjects were not allowed to talk, laugh, move or sleep while lying under the hood [17].

**Table 1:** Ingredients per capsule (mg).

	GT	PL
**Total polyphenols**	207.1	-
*** Total catechins***	*169.0*	-
** ** ***Epigallocatechin gallate***	*84.5*	-
**Caffeine**	4.2	-
**Soy oil**	316.9	528.2
**Total filling weight**	528.2	528.2
**Total weight capsule**	757.0	757.0

PL, placebo; GT, green tea; GT: Sunphenon 90 LB (Taiyo Kagaku Co. Ltd, Mie, Japan) decaffeinated green tea extract. Subjects received three capsules per test day.

### DNA isolation and genotyping

Blood was collected in an EDTA tube during screening and the buffy coat was stored at −80°C. Genomic DNA was isolated from the buffy coat using the QIAamp mini blood kit (Qiagen, Amsterdam, The Netherlands). Genotyping of the *Val(108/158)Met* polymorphism of the COMT gene (RS4680) was performed using a commercially available TaqMan SNP genotyping assay from Applied Biosystems (Foster City, California, USA). The procedure was performed according to the manufacturer’s protocol and measured on an Applied Biosystems 7900 HT Fast Real-Time PCR system. Allelic calls were determined semi-automatically using the allelic discrimination software of Applied Biosystems.

### Statistical analysis

Data are presented as means ± standard errors, unless otherwise indicated. A Chi-square test was used to check whether the genotype frequencies were in Hardy Weinberg equilibrium. A one-way ANOVA was used to assess possible differences with respect to subject characteristics between genotypes, at baseline. A two-factor ANOVA with genotype (COMT^H^ vs COMT^L^) as factor 1 and treatment (GT vs PL) as factor 2 was used to assess differences between genotype and treatment, as well as a possible interaction effect. Pairwise comparisons allowed us to locate the significant differences between treatments or between genotypes. Also, deltas (GT-PL) were calculated to compare the relative changes between COMT genotypes, which emphasize the interaction between COMT genotypes and treatment. A one-way ANOVA was used to assess possible differences for subject characteristics between genotypes. Data were analyzed using PASW Statistics 18.0 (SPSS Inc. Chicago, Illinios, USA). The level for establishing significant differences was taken at p<0.05.

## Results

**Figure 1:**
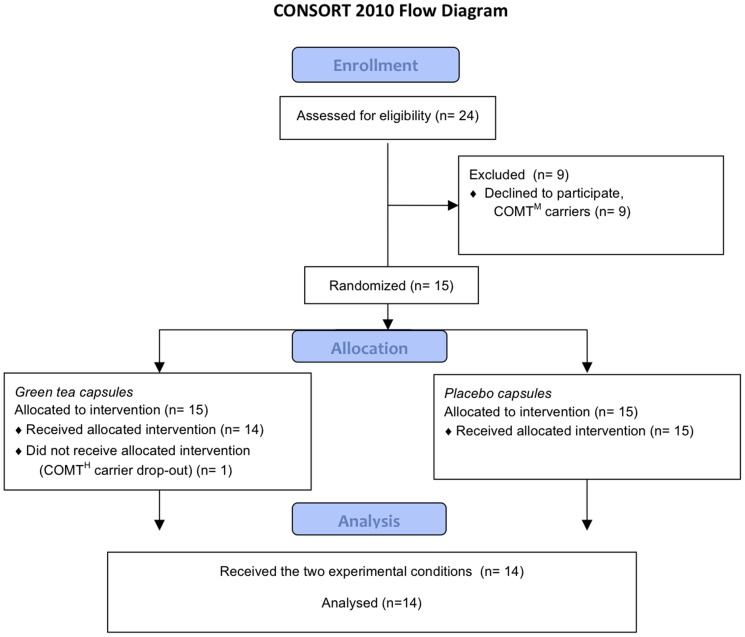
Flow diagram (CONSORT).

Fourteen Caucasian subjects (8 females, 6 males) participated in this study, they were healthy, aged 19 to 27 years and had a BMI between 18 and 26 kg/m^2^ (**Table 2**). From the initial 24 volunteers, one COMT^H^ carrier withdrew from the study without giving any reason. Also, 9 subjects carried the intermediate-activity COMT^M^ genotype and were therefore not included in this study (**Figure 1**). No adverse events occurred as no subject reported any discomfort after consuming the capsules. No different effects between men or women were observed therefore these data have been pooled together. The single nucleotide polymorphism of the COMT genotype was in Hardy Weinberg equilibrium (**Table 3**).

**Table 2:** Subject characteristics.

	Total	COMT^H^	COMT^L^	*P-values*
N (M/F)	14 (6/8)	7 (4/3)	7 (2/5)	
Age (year)	22.0±2.3	22.9±2.7	21.1±1.5	0.193
Height (m)	1.76±0.08	1.76±0.09	1.75±0.09	0.814
Body weight (kg)	69.3±11.3	71.4±7.8	67.1±14.4	0.284
BMI (kg/m^2^)	22.4±2.6	23.0±2.1	21.7±3.1	0.176
FM (kg)	15.3±6.5	16.8±3.6	13.8±8.5	0.257
FFM (kg)	54.0±6.6	54.6±7.5	53.3±6.1	0.703

COMT^H^, high activity catechol-O-methyl transferase genotype; COMT^L^, low activity catechol-O-methyl transferase genotype; BMI, body mass index; FM, fat mass; FFM, fat free mass. Values are means ± standard deviations. Data were analyzed with a one-way ANOVA.

**Table 3:** Genotypic and allelic distribution.

Gene	SNP	G	F (N)	F (%)	Allele	F (%)	HWE
*COMT*	rs4680	GG *(Val/Val)*	8	33.3	G	52.1	0.22
		GA *(Val/Met)*	9	37.5	A	47.9	
		AA *(Met/Met)*	7	29.2			

SNP, single nucleotide polymorphism; G, genotype; F, frequency both absolute (N) and relative (%); COMT, catechol-O-methyl transferase. P-value obtained from the χ^2^-test of Hardy Weinberg equilibrium.

**Figure 2:**
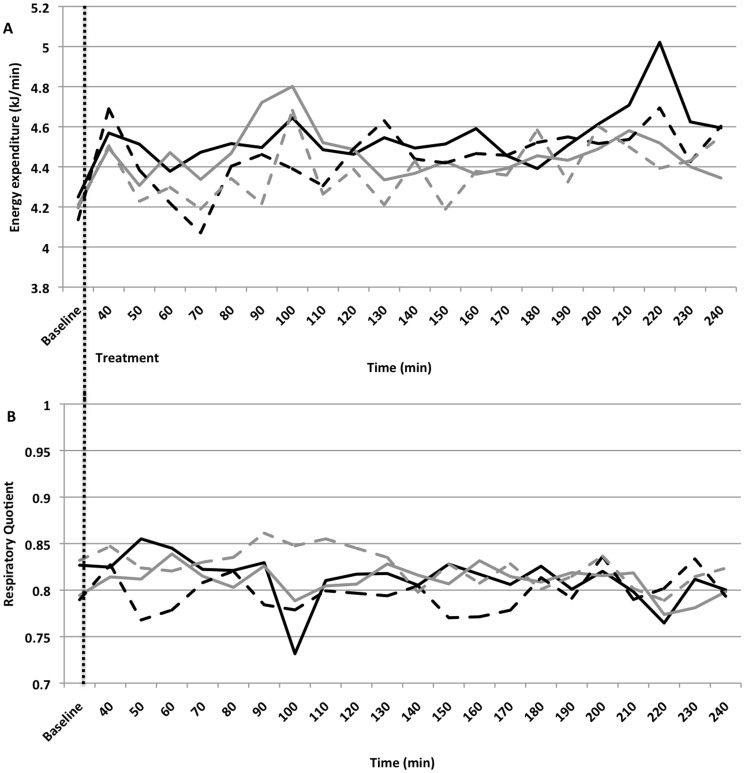
Real-time energy expenditure (EE; kJ/min, 2A) and RQ (2B) before and after consumption of green tea (Black line) and placebo (Grey line) capsules in subjects carrying a COMT^L^ genotype (Solid line) and COMT^H^ genotype (Dotted line). Baseline resting energy expenditure and RQ were measured during the first 30 minutes. The vertical dotted line indicates the time that the treatment was given. Values are means (two-factor ANOVA with genotype as factor 1 and treatment as factor 2).

At baseline, EE (**Figure 2A**), RQ (**Figure 2B**), FATox and CHOox did not differ between genotypes, neither did they differ between treatments (**Table 4**). Significant interactions were observed between COMT genotypes and treatment for RQ, FATox and CHOox (p<0.05) (**Table 5**).

**Table 4:** Baseline values of measured variables.

			*P-values*			*P-values*
	GT	PL		COMT^H^	COMT^L^	
EE (kJ/min)	4.19±0.53	4.20±0.65	0.910	4.17±0.68	4.23±0.77	0.872
RQ	0.81±0.03	0.81±0.03	0.786	0.81±0.03	0.81±0.02	0.983
FATox (g/min)	17.1±4.4	16.5±4.9	0.558	16.9±1.3	17.0±1.2	0.994
CHOox (g/min)	20.4±4.1	22.1±6.3	0.684	21.4±0.9	21.4±2.0	0.963

EE, energy expenditure; RQ, respiratory quotient; FATox, fat oxidation; CHOox, carbohydrate oxidation; GT, green tea; PL, placebo; COMT^H^, high activity catechol-O-methyl transferase genotype; COMT^L^, low activity catechol-O-methyl transferase genotype. Values are means ± standard errors. Two-factor ANOVA with genotype (COMT^H^ vs COMT^L^) as factor 1 and treatment (GT vs PL) as factor 2.

**Table 5:** Results for measured variables.

		EE (kJ/min)	RQ	FATox (g/min)	CHOox (g/min)
ALL	GT	61.3±6.6	0.80±0.02	17.8±0.5	20.3±0.9
	PL	43.6±6.0	0.82±0.02	16.2±0.4	22.8±0.7
ALL	COMT^H^	48.8±5.8	0.81±0.02	16.8±0.4	21.4±0.8
	COMT^L^	56.0±6.7	0.81±0.01	17.2±0.6	21.7±1.0
COMT^H^	GT	62.2±8.0	0.80±0.02	18.3±0.5	18.5±1.2
	PL	35.4±7.7	0.83±0.02	15.3±0.5	24.3±1.1
COMT^L^	GT	60.3±7.4	0.81±0.03	17.3±0.5	22.1±1.4
	PL	51.7±6.7	0.81±0.02	17.0±0.5	21.4±0.9
		*P-values*	*P-values*	*P-values*	*P-values*
Treatment	GT	0.006	0.004	0.001	0.011
	PL				
Genotype	COMT^H^	0.259	0.958	0.452	0.727
	COMT^L^				
Treatment*genotype		0.154	0.001	0.005	0.001
				

EE, energy expenditure; RQ, respiratory quotient; FATox, fat oxidation; CHOox, carbohydrate oxidation; GT, green tea; PL, placebo; COMT^H^, high activity catechol-O-methyl transferase genotype; COMT^L^, low activity catechol-O-methyl transferase genotype. Values are means ± standard errors. Two-factor ANOVA with genotype (COMT^H^ vs COMT^L^) as factor 1 and treatment (GT vs PL) as factor 2.

Also, when comparing the difference between GT and PL for genotypes, the delta was significantly larger in the COMT^H^ genotype vs. COMT^L^ genotype for EE (26.8±8.8 vs. 8.63±8.6 kJ.3.5 hrs; p<0.05), RQ (−0.03±0.03 vs. 0.002±0.02; p<0.001), FATox (3.00±0.7 vs. 0.24±0.5 grams/d; p<0.01) and CHOox (−5.76±1.5 vs. 0.68±1.2 grams/d; p<0.001).

For treatment, significant differences were observed on EE, RQ, FATox and CHOox. In COMT^H^ and COMT^L^ groups combined, GT vs. PL increased EE (61.3±6.6 vs. 43.6±6.0 kJ.3.5 hrs; p<0.01) and FATox, (17.8±0.5 vs. 16.2±0.4 g/d; p<0.01), while RQ (0.80±0.02 vs. 0.82±0.02; p<0.01) and CHOox (20.3±0.9 vs. 22.8±0.7 g/d; p<0.03) were decreased.

**Figure 3:**
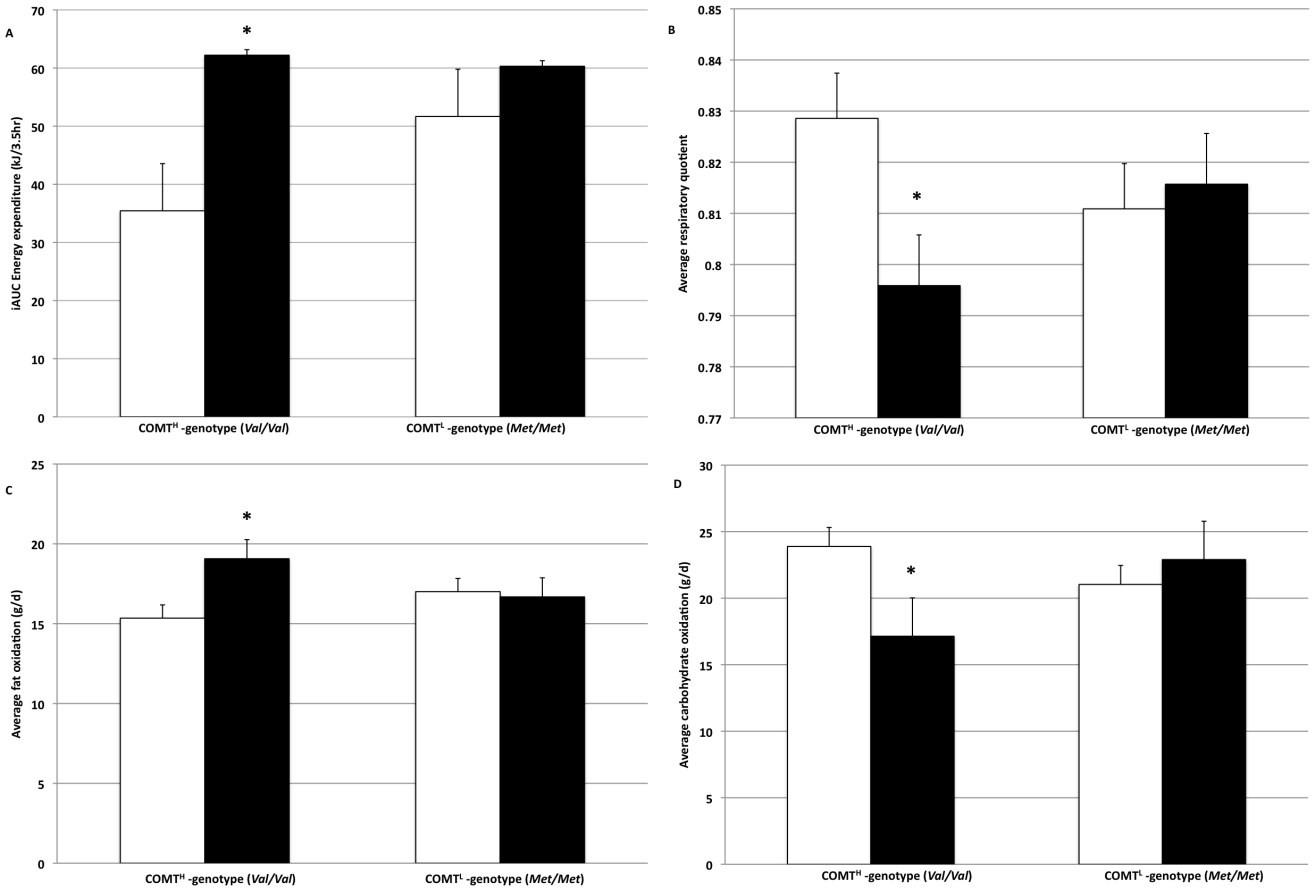
Results of the incremental area under the curve (iAUC) for energy expenditure (3A; p<0.01), average respiratory quotient (3B; p<0.01), average fat oxidation (3C; p<0.001) and average carbohydrate oxidation (3D; p<0.001) in the green tea (black) and placebo (white) conditions in fourteen subjects carrying either a COMT^H^ genotype or COMT^L^ genotype. Values are means, with standard errors represented by vertical bars. *Mean values for green tea were significantly different from placebo condition (two-factor ANOVA with genotype as factor 1 and treatment as factor 2).

For subjects carrying the COMT^H^ genotype, EE (GT: 62.2±8.0 vs. PL: 35.4±7.7 kJ.3.5 hrs; p<0.01) (**Figure 3A**) and FATox (GT: 18.3±0.5 vs. PL: 15.3±0.5 grams/d; p<0.001) (**Figure 3C**) were significantly elevated after GT vs. PL, but not for subjects carrying the COMT^L^ genotype (EE, GT: 60.3±7.4 vs. PL: 51.7±6.7 kJ.3.5 hrs; NS), (FATox, GT: 17.3±0.5 vs. PL: 17.0±0.5 grams/d; NS). Correspondingly, RQ (GT: 0.80±0.02 vs. PL: 0.83±0.02; p<0.01) (**Figure 3B**) and CHOox (GT: 18.5±1.2 vs. PL: 24.3±1.1 grams/d; p<0.001) (**Figure 3D**) were significantly decreased after GT vs. PL for subjects carrying the COMT^H^ genotype, but not for subjects carrying the COMT^L^ genotype (RQ, GT: 0.81±0.03 vs. PL: 0.81±0.02; NS), (CHOox, GT: 22.1±1.4 vs. PL: 21.4±0.9 grams/d; NS).

## Discussion

After investigating the effect of green tea and placebo consumption in subjects carrying different COMT genotypes, the results show that genetic predisposition may play an important role in whether or not subjects benefit from green tea. Significant interactions between treatment and COMT genotypes were observed for substrate oxidation. Also, the difference between GT and PL was much larger in the COMT^H^ genotype carriers compared with the COMT^L^ genotype carriers for EE, RQ, FATox and CHOox, which suggests a larger response of the COMT^H^ genotype after GT ingestion. After treatment, it was observed that the impact of GT was more present in COMT^H^ genotype carriers. These show a significant increase in EE and FATox as well as a significant decrease in RQ and CHOox that did not occur in the COMT^L^ genotype carriers. Between genotypes, RQ and CHOox were significantly lower in the COMT^H^ genotype carriers compared with the COMT^L^ genotype carriers after GT vs. PL consumption. These findings may explain the variability as observed between subjects in response to GT administration, as well as the inconsistent results between studies investigating the beneficial effects of GT. It also shows that genetic predisposition may be a moderating factor as previously hypothesized in several manuscripts. The current results support the conclusions of a meta-analysis [10] that showed a seemingly smaller effect of catechins in Caucasian (−0.82 kg; 95% CI: −2.13 to 0.50) subjects compared with Asian subjects (−1.51 kg; 95% CI: −2.37 to −0.65) (21). Jurgens et al. [11] drew a similar conclusion from their meta-analysis.

Remarkably, the interaction that we observed was partly due to subjects carrying the COMT^L^ genotype having a higher PL induced EE and FATox, yet not an elevated GT induced EE compared with subjects carrying the COMT^H^ genotype. Contrarily, the COMT^H^ genotype carriers hardly increased EE and FATox after PL ingestion, while they did increase EE and FATox significantly after GT ingestion. Nevertheless, based on the previous, it may well be that studies including mostly subjects with the COMT^H^ genotype, such as Asians, lead to more significant results by measuring a response which is not or less present in Caucasians that are more likely to be COMT^L^ genotype carriers. This response may be modulated by SNS activity since COMT is a noradrenalin-degrading enzyme, with most likely different degrading rates between genotypes. However, methylation of catechins by COMT is not the only step in the conjugation process that precedes absorption. Glucuronidation and sulfation could potentially be susceptible to green tea catechins in a similar way as methylation. Phase II enzymes involved in these processes may lead to different outcomes as shown in studies examining the absorption and bioavailability of catechins. These are in line with intervention trials that report similar inconsistent results regarding the presence of metabolites in urine and plasma [18], [19]. Miller et al. [20] examined the effect of COMT genotype on absorption and metabolism of catechins and concluded that different polymorphisms seem to have no large impact. In contrast, Choi et al. [21] demonstrated in 660 daily GT drinking subjects that subjects with the low-activity COMT genotype excreted less catechin metabolites via their urine compared with subjects that carried the high-activity genotype. The absence of an effect in the study of Miller et al. [20] was attributed to the low availability of catechins. This was explained by the existence of two different COMT proteins; cytoplasm soluble protein (S-COMT) and membrane bound protein (MB-COMT). It appears that S-COMT has more affinity for metabolizing catechins, while MB-COMT metabolizes catecholamines. Nevertheless, it is debatable whether this makes any difference as S-COMT is the predominant form in most tissues and responsible for the majority of COMT enzyme activity, whereas only a small part of activity can be attributed to MB-COMT [22].

Moreover, Nackley et al. [23] suggested that, beside the *Val(108/158)Met* polymorphism, there are additional polymorphisms in the COMT gene that modulate enzyme activity. Four polymorphisms in the COMT gene have been demonstrated to combine into 3 common haplotypes [24], which have been associated with variation in COMT enzyme activity [23]. It should be taken into consideration that haplotype may account more for variability than an individual polymorphism and, therefore, play an important role in the effect of GT on EE and substrate oxidation.

Finally, it should be mentioned that the GT capsules contained a small amount of caffeine, which was not present in the PL capsules. Although it is not likely to have influenced the current results it should be taken into consideration. Usually, GT contains caffeine and the metabolism of caffeine may depend on the enzyme activity of *CYP1A1-CYP1A2* gene, which also differs between individuals [25].

Summarizing, subjects carrying the COMT^H^ genotype may respond with increases of EE and FATox upon GT vs. PL ingestion, whereas COMT^L^ genotype carriers react similarly to GT and PL ingestion. Green tea catechins appear to compensate for higher noradrenalin degradation in the COMT^H^ genotype by inhibiting COMT. However, whether genetic predisposition is also a moderator in the long-term is still not evident, warranting a large-scale study in different ethnic populations. Also, interactions of different *CYP1A1-CYP1A2* and *COMT* polymorphisms as well as the role of haplotypes in the effect of GT on EE and substrate oxidation should be studied in the future.

In conclusion, subjects carrying the COMT^H^ genotype increased energy expenditure and fat-oxidation upon ingestion of green tea catechins vs, placebo, whereas COMT^L^ genotype carriers reacted similarly to GT and PL ingestion. The differences in responses were due to the different responses on PL ingesting, pointing to different mechanisms. The different alleles of the functional COMT *Val108/158Met* polymorphism appear to play a role in the inter-individual variability for EE and FATox after GT treatment.

## Supporting Information

Protocol S1 **Study protocol containing background, hypothesis, outcome parameters and experimental design.**
(PDF)

Checklist S1 **CONSORT checklist.**
(DOC)
