# Supplementary material for: The Role of Catechol-O-Methyl Transferase Val(108/158)Met Polymorphism (rs4680) in the Effect of Green Tea on Resting Energy Expenditure and Fat Oxidation: A Pilot Study
Source: PLoS One. 2014 Sep 19;9(9):e106220. doi: 10.1371/journal.pone.0106220 (PMC4169515; doi:10.1371/journal.pone.0106220)
Supplement: Protocol S1 — Study protocol containing background, hypothesis, outcome parameters and experimental design. (PDF) [file pone.0106220.s001.pdf]

- Research proposal -

The role of genetic predisposition in the effect of green tea on fat oxidation and energy expenditure

R Hursel and MS Westerterp-Plantenga

Corresponding author:

R Hursel
Maastricht University
Dept. Human Biology
PO Box 616
6200 MD Maastricht
Phone: +31 43 3884596
E-mail: rick.hursel@HB.unimaas.nl

April 2009, third version

Table of contents

Introduction…………………………………………………….. 4
Objectives…………………………………………………… 6
Subjects……………………………………………………… 6
Study design………………………………………………… 6
Sample size…………………………………………………. 7
Methods and measurements……………………………… 7
Screening…………………………………………... 7
Protocol………………………………………........ 8
Indirect calorimetry………………………………… 8
Energy expenditure and substrate oxidation……. 9
Fat oxidation………………………………………… 9
Urine collection……………………………………… 10
Energy intake……………………………………... 10
Questionnaires……………………………………… 10
Information about capsules……………………….. 10
Outcome parameters………………………………………. 11
Statistics……………………………………………………… 11
Safety and risks……………………………………………… 12
Ethics………………………………………………………….. 12
Publication…………………………………………….......... 13
Study planning……………………………………………….. 13
List of abbreviations………………………………………….. 14
References……………………………………………………. 15

Summary

Research title
The role of genetic predisposition in the effect of green tea on fat oxidation and energy expenditure are

Background
Recent epidemiological studies have suggested that the effect of green tea on fat oxidation and energy expenditure might differ because of differences in the genetic predisposition. This may largely be caused by allele frequencies of relevant polymorphisms that play a major role within the subjects. The effect of green tea is induced by a combination of active components of green tea, the catechins that inhibit the enzyme catechol-O-methyl transferase (COMT) and caffeine that inhibits the enzyme phosphodiesterase (PDE). COMT normally degrades norepinephrin and PDE degrades cAMP. Both increase sympathetic nervous system activity, which will occur continuously after the inhibition of both enzymes thereby resulting in an elevated fat oxidation and energy expenditure. For COMT, three polymorphisms are known from which the *Val/Val* polymorphism codes for the $COMT^{H}$ allele, the thermo stabile enzyme with high activity; the *Met/Met* polymorphism codes for the $COMT^{L}$ allele, the thermo labile enzyme wit low activity; and the *Val/Met* polymorphism that codes for the $COMT^{H/L}$ allele, the enzyme with intermediary activity.
Asians appear to be mainly predisposed for the $COMT^{H}$ allele (approximately 75%). From the Caucasians only a minority possesses this polymorphism (25%) since the majority is predisposed with the $COMT^{H/L}$ allele (50%). Therefore, the success rate for lowering body weight through the effect of green tea on fat oxidation and energy expenditure might be increased in Asians compared with Caucasians.

Objective
Investigating the role of genetic predisposition in the effect of green tea on:
- (i) energy expenditure and substrate metabolism
- (ii) hunger and satiety, and energy intake.

Study design
The study has a randomized, crossover design with two conditions. Randomization of the conditions will take place with the help of a digital randomization program.

Population
Forty-eight healthy subjects with a BMI between 18-25 kg/m^2 and aged between 18-50 years from which 24 Caucasians (12 with the $COMT^{H}$ allele and 12 with the $COMT^{L}$ allele) and 24 Asians (12 with the $COMT^{H}$ allele and 12 with the $COMT^{L}$ allele).

Outcome parameters
The outcome of the study will be the effect of green tea on energy expenditure and fat oxidation in subjects that have different COMT polymorphisms, measure din a respiratory chamber. Furthermore, the effect of green tea on hunger and satiety feelings will be determined in the same subjects.

Description of the burden and risks for the subjects
The study contains 2 x 2 test days with a total duration of 72 hours. The subjects will be in the chambers for the total 36 hours stay. There are no risks attached for the subjects while participating in this study.

Applications

The outcome of the study can be used for a more individual approach for weight management. Besides green tea more bioactive ingredients that are currently investigated for their weight reducing capabilities may be considered for a specific diet, when their has been established if there are responders and non-responders because of different polymorphisms. Also, it gives an indication whether green tea is an efficient tool for weight management.

Introduction

Overweight and obesity represent a rapidly growing threat to the health of populations in an increasing number of countries [1]. The ultimate cause of obesity is an imbalance between energy intake and energy expenditure (EE)[2]. A negative energy balance is needed to produce weight loss and can be achieved by either decreasing intake or increasing expenditure. Classical weight loss programmes, such as low-fat diets, behavioral modification and exercise, often fail to achieve a long-term maintenance of weight loss [3, 4]. Because of these low success rates, the stimulation of EE (or the prevention of its decline during dieting) by the use of natural herbal nutrients such as green tea and caffeine has attracted interest.

Green tea (GT) is consumed primarily in China, Japan and a few countries in North Africa and the Middle East [5, 6]. Tea is made from the leaves of Camellia sinensis L. species of the Theaceae family, GT being the non-oxidised, non-fermented product. As a consequence of this, it contains high quantities of several polyphenolic components such as epicatechin, epicatechin gallate, epigallocatechin and, the most abundant and probably the most pharmacologically active, epigallocatechin gallate (EGCG) [7]. From caffeine, that is also present in GT, it has been reported that it has thermogenic effects and can stimulate fat oxidation in vitro, in part via sympathetic activation of the central nervous system [8]. In humans, caffeine has been shown to stimulate thermogenesis and fat oxidation [9-11]. GT extracts, containing caffeine and catechin-polyphenols, have been reported to have an effect on body weight [7, 12] and EE [13, 14]. The fact that GT stimulates thermogenesis cannot be completely attributed to its caffeine content because the thermogenic effect of GT is greater than that of an equivalent amount of caffeine [13]. As GT combines the best of catechins and caffeine, it has already shown in the past to be an interesting thermoactive food ingredient that assists in preventing or treating overweight in normal weight to obese people.

The sympathetic nervous system (SNS) plays an important role in the regulation of energy homeostasis. Catechins inhibit the enzyme catechol-O-methyl transferase (COMT) which usually degrades norepinephrine. Levels of this neurotransmitter rise in the synaptic cleft as it is not degraded anymore. The activity of the SNS increases and via the elevation of adenyl cyclase subsequently levels of cyclic amino mono phosphate (cAMP), and phospho kinase A (PKA) rise. Consequently, glucose uptake is decreased, lipolysis and thereby fat oxidation is stimulated and uncoupling proteins are upregulated resulting in thermogenesis. Caffeine exerts its effect by inhibiting the

enzyme phosphodiesterase (PDE), that usually degrades cAMP to AMP thereby activating the same pathway as the catechins [15]. An additional advantage is that caffeine binds to the A2a receptor, thereby preventing the binding of adenosine, that is able to decrease norepinephrine, to these receptors, The increase in energy expenditure and fat oxidation does not always appear equally clear in all ethnic groups. For instance studies with Asian subjects seem to report more positive results than studies with Caucasian subjects. Recent literature suggests that this may be caused by differences in relevant enzyme activity, causing differences in sensitivity for these ingredients. In that respect Hodgson *et al.* [16] stated that there is a wide variability in flavonoid O-methylation, a major pathway of flavonoid metabolism, by the enzyme COMT. The inter-individual variability of the activity of COMT could vary as much as 3-fold [16]. Moreover, there is evidence that there is a difference in COMT enzyme activity between ethnic groups [17]. Asian populations have a higher frequency of the thermostable, high activity enzyme, $COMT^H$ allele (*Val/Val* polymorphism) than the Caucasian populations. The Caucasian populations have a higher frequency of the thermolabile, low activity enzyme, $COMT^L$ allele (*Met/Met* polymorphism) [17]. 50% of Caucasians are homozygous for the $COMT^L$ allele (25%) and $COMT^H$ allele (25%). The other 50% is heterozygous (*Val/Met* polymorphism) [17]. This may explain the difference in sensitivity to interventions with green tea caffeine mixtures, and why, in some studies with Caucasian subjects, no effect was seen after ingestion of green tea.

We hypothesize that subjects (Asian or Caucasian) with the $COMT^H$ allele (*Val/Val* polymorphism) respond stronger to the GT treatment than subjects with the $COMT^L$ allele (*Met/Met* polymorphism), consequently have an increased EE and that the genetic differences between Asians and Caucasians are responsible for the different outcomes in GT studies with both subjects.
Therefore, the aim of the present study is to reveal whether genetic predisposition plays a role in the effect of green tea on energy expenditure and fat oxidation, by measuring Caucasian as well as Asian subjects with either a high or low activity polymorphism for the enzyme COMT. This study might reveal why green tea has an effect only in certain people and whether it is only a useful method in these people to initiate weight loss or prevent weight gain.

Objectives

The objectives of this study are to determine the effect of the different COMT polymorphisms after ingestion of green tea on energy expenditure and fat oxidation in a respiration chamber when being fed in energy balance.

Subjects

Fourty-eight healthy subjects (age 18-50 years and BMI 18-25 kg/m^2) from which 24 Caucasians and 24 Asians, from each ethnicity 12 with a COMT^H allele and 12 with a COMT^L allele, will be recruited. The subjects will be recruited via advertisements in the University building and in a local newspaper. Subjects have to be in good health, non-smokers, not using medication (except the use of contraceptive), at most moderate alcohol users (persons consuming less than 10 glasses/week) and also people who drink at most one cup of coffee per day. Dulloo et al. and Kovacs et al. showed that the effect of green tea administration was the highest in people who drunk less than 2 cups of coffee per day [13, 18]. During the initial screening 10 ml blood will be drawn from the subjects to determine the polymorphism. We choose to draw blood instead of taking a sample from the mucous membrane of the cheek as the latter results in 10-20% drop-outs during the analysis. This would mean that more subjects have to be screened or that subjects have to be screened more than once, in case the analyses fail which is more aggravating for the subjects than drawing 10 ml blood once. A questionnaire on health, use of medication, smoking behaviour, alcohol consumption and tea and coffee consumption will be completed. The Dutch translation of the Three Factor Eating Questionnaire (TFEQ) will be used to determine eating behaviour [19, 20]. Non-restrained eaters (<9 times factor 1), these are persons who are not consciously occupied with food and who are caloric restricted, will be selected.

Study design

The study will have a randomized, single-blinded, cross-over design. Randomization takes place using a computerized randomization program. Only the subjects are blinded, as the researcher has to supply the subjects with the treatment. Blinding the researcher as well is not possible because there is no option to check afterwards which capsule the subjects consumed.

Sample size

The primary parameter is total energy expenditure. Dulloo *et al.* [13] report TEE values of 9867 ± 488 kJ/day and 9538 ± 521 kJ/day for green tea vs placebo [13]. With an α of 0.05 and β of 0.10 (power=1-β=0.90) the number of subjects needed is:

$$N=21x(SD)^2/(mean1-mean2)^2+0.96$$

$$N=21x(488)^2/(9867-9538)^2+0.96$$

= 47 subjects needed

Rounded up, 48 subjects will be included in the experiment which means that each groups contains 12 subjects. When taking drop outs into account 193 persons need to be screened. For the Asians with the $COMT^H$ allele 17 people have to be screened to get 12 subjects, as the allele frequency is 70%. The allele frequency for the $COMT^L$ allele is 15%, which means that 80 people have to be screened. In Caucasians for either the $COMT^L$ allele as well as for the $COMT^H$ allele 48 people have to be screened, as the allele frequencies are 25%. In total this would mean that 193 subjects have to be screened in order to find 48 subjects. This is an obtainable number since habitual high coffee drinkers do not apply for entering the study as the poster and advertisements mention the inclusion criteria. Also, coffee consumption in Asian people is rather low compared with Caucasians.

An ANOVA repeated measures with post-hoc tests will be used to compare the effects of green tea on TEE and fat oxidation in subjects with different ethnicity and different COMT polymorphisms. A p-value <0.05 will be regarded as statistically significant.

Methods and measurements

Screening

During screening, which takes place in our lab at the university, height and body weight will be determined. A questionnaire on health, use of medication, smoking behaviour and alcohol, coffee and tea consumption will be completed as well as a questionnaire to characterize eating behaviour (TFEQ). The outcome of the questionnaire will give an indication whether a subject is in good health or not as they have to report their use of medicine, disease history of themselves and their family. Finally, healthy, non-smoking males and females aged 18-50 years and with a BMI ranging from 18 till 25 kg/m^2 that have either no food allergies, are non-restrained eaters, moderate alcohol consumers and low habitual coffee drinkers will

be included in this study. Gender has no effect on the outcome, therefore both men and women are included. The age range is determined in order to obtain a vast range of subjects without emphasis on effects of aging. Only adults will be included in the experiment and therefore it is chosen to take an age of 18 as a limit. Subjects older than 50 years are excluded as they might respond less to treatment than younger subjects (for instance menopausal women). The BMI range concerns normal subjects and has been chosen because we first want to test the hypothesis in this group. Testing the hypothesis in obese subjects will be the next step. Also, green tea can be used to prevent weight gain in the non-obese group. During the initial screening of the subjects 10 ml blood will be drawn to determine their polymorphism. The screening will last approximately 30 minutes and will not be compensated. Eventually the outcomes of the questionnaires and the analysis of the blood sample will give us an indication whether the subject is suitable for the study. Subjects do not have to prepare for this screening.

Protocol

Subjects will visit the university twice and during each visit they will stay in the respiration chamber for two days and nights (36hrs). During one of these visits subjects will test the treatment condition (green tea; 757 mg/capsule [84.5 mg EGCG + 2.1 mg caffeine], 3 capsules with each breakfast and dinner) and the other visit they will test the placebo condition (soy oil; 757 mg/capsule, 3 capsules with each breakfast and dinner). Subjects receive a glass of water in order to swallow the capsules more comfortably. Both visits are separated by a period of 4 weeks, which will prevent influences of the previous experiment and influences of differences in menstrual cycle phase on energy expenditure. The respiration chamber experiment starts in the evening of day 1 at 19:00h and ends at 07:00h on day 3.

Indirect calorimetry

Oxygen consumption and carbon dioxide production will be measured in the respiration chamber [21]. The respiration chamber is a 14 m^3 hotel room, furnished with a bed, chair, computer, television, radio, dvd-player, telephone, intercom, sink and toilet. The room is ventilated with fresh air at a rate of 70-80 l/min. The ventilation rate is measured with a dry gas meter. The concentrations of oxygen and carbon dioxide will be measured using a paramagnetic O_2 analyzer and an infrared CO_2 analyzer. During each 15-min period six samples of outgoing air for each

chamber, and one sample of fresh air, zero gas, and calibration gas will be measured. The gas samples to be measured will be selected by a computer that also stores and processes the data [21].

Energy expenditure and substrate oxidation

24-h energy expenditure consists of sleeping metabolic rate (SMR), diet-induced energy expenditure (DEE), and activity-induced energy expenditure (AEE). 24-h energy expenditure and 24-h respiratory quotient (RQ) will be calculated from 19:00h on day 1 to 07:00h on day 3, using the formula of Brouwer [22]. Physical activity is monitored using a radar system working on the Doppler principle. SMR is defined as the lowest mean energy expenditure measured over three consecutive hours between 00:00h and 07:00h. DEE will be calculated by plotting energy expenditure against radar output, both averaged over 30-min periods. The intercept of the regression line at the lowest radar output represents the energy expenditure in the inactive state (Resting Metabolic Rate; RMR), consisting of SMR and DEE [23]. DEE will be determined by subtracting SMR from RMR. Activity-induced energy expenditure will be determined by subtracting RMR from 24-h energy expenditure. The Physical Activity Level (PAL) will be calculated by dividing 24-h energy expenditure by SMR. Carbohydrate, fat, and protein oxidation will be calculated using O_2 consumption and CO_2 production, deuterium labelled palmitic acid and urinary nitrogen excretion, with the formula of Brouwer [22].

Fat oxidation

Deuterium labelled palmitic acid (Cambridge Isotope, Andover, MA): d31-palmitic acid (DLM-215) was 98 atom%. This palmitic acid (20mg/kg body weight) is added to tomato juice and consumed at breakfast. Usually chocolate milk is used but recently we have published that the effect of green tea is not always present in combination with milk [24]. Tomato juice contains catechins as well, although in low amounts. The main antioxidant in tomato is besides vitamin C, lycopene. This antioxidant has just like catechins from green tea a beneficial effect on cancer. It does not affect energy expenditure and substrate oxidation such as the catechins from green tea. There is no EGCG, which is the most active form in green tea, present in tomatoes [25]. Therefore tomatojuice will not influence the results. Also, when the juice is given in both conditions the difference in outcome parameters between both conditions remains the same. When subjects dislike tomato juice a different juice can be

selected for dissolving the palmitic acid. Urine samples will then be collected every 2 hours from 07.00h day 2 till 19.00h day 2 [26]. The product information for the palmitic acid can be found in the attachments. It is a product that is suitable for human consumption as it is already used in several studies [27-29], also in a previously by the medical ethical committee approved study (MEC 06-3-051).

Urine collection

Urine will be collected from 19.00h day 1 till 07.00h day 2, 07.00h day 2 till 19.00h day 2, and 19.00h day 2 till 07.00h day 3. Samples will be collected in containers with 10 ml H_2SO_4 to prevent nitrogen loss through evaporation. The subjects will be told, before entering the respiration chamber, to handle the urine containers with the most caution because of the presence of H_2SO_4. Volume and nitrogen concentration will be measured, the latter using a nitrogen analyzer.

Energy intake

During their stay in the chamber subjects will be fed in energy balance. Subjects receive their breakfast, lunch and dinner, at regular time points. The energy content of the food will be calculated using the equation of Harris-Benedict [30] that calculates Basal Metabolic Rate (BMR). BMR will be multiplied by an activity index of 1.35. Energy intake will be divided over the meals as 20% for breakfast, 40% for lunch, and 40% for diner. The macronutrient composition of the diet will be 12/55/33 En% (protein/carbohydrate/fat). The diet will consist of normal food products, available in the supermarket. The actual diet will be determined in consultation with the subjects. They can indicate which food products they dislike and based on this information a diet will be composed with the abovementioned macronutrient composition. All foods will be prepared in a research kitchen from the Department of Human Biology by one of the researchers.

Questionnaires

The appetite profile will be scored every hour by the following anchored 100 mm Visual Analog Scales: How hungry/satiated/full/thirsty are you? (Anchors: not at all; very much); How is your desire to eat? (Anchors: very little; very large) [31].

Information about capsules

The product information for the green tea capsules can be found in the attachments.

For the placebo capsules there is no direct product information available, the product information from Gelkaps is mend for placebo and green tea capsules. When specifying the placebo capsules the green tea extract is replaced with soy oil. The placebo thereby consists for 100% of soy oil. Soy oil is a globally consumed plant oil, and often used as a food additive and has hardly any flavour. Lecithin, which is also present in very small amounts in the capsules, is a product of soy oil after processing. It works as an emulsifier. These capsules have already been used in a previous study (NL19702.068.07/ MEC 07-3-080) and been approved by the medical ethical committee.

Outcome parameters

Endpoints of the study are:
Energy expenditure, substrate oxidation, hunger and satiety in subjects who differ in genetic polymorphism and /or in ethnicity. More detailed:

- Effect of green tea on total energy expenditure and substrate oxidation, measured in the respiration chamber
- Feelings of hunger and satiety measured with VAS
- Effect of the different polymorphisms in people with different ethnicities on the outcome of energy expenditure and substrate oxidation after supplementation of green tea

For further information see paragraphs above.

Statistics

The average of each group in each of the 2 conditions (green tea or placebo) is calculated for:

- Mm VAS at each time-point; the area under the curve (AUC) is calculated for hunger, satiety, fullness, thirst, desire to eat, time of desire of next meal. This is done with the trapezoid method.
- Conditions are compared using 4-factor ANOVA repeated measures with post-hoc tests to discriminate between groups, and will show possible differences in satiety and energy expenditure. To double-check the outcomes, paired sample T-tests will be performed as well.
- Comparison implies the clinical outcome parameters as mentioned in the paragraph above.
- All results will be visualized in graphs and tables for better understanding.

Safety and risks

This study does not include any risk for the subjects. There are no risks for the subjects in using any of the meals because people with certain food allergies are excluded. Blood sampling during the screening is limited and without side effects, apart from its usual risks of minor bruising. Green tea is a natural product that has no side effects, catechins can only be toxic in extreme dosages that can not be reached by drinking tea. Caffeine is only lethal after approximately 10 grams, which equals more than 100 cups of coffee. The dosage used here for green tea is based on the study of Dulloo et al.[13] who conducted several trials with different dosages and found that the current dose was ideal. Berube-Parent et al. [32] who conducted a study with different dosages confirmed this, as they found no effect of increasing dosage. The deuterium and palmitic acid (a regular fatty acid) are also not dangerous as both are freely available in nature and food products. The body will process them comparable as when taken up via food products. They contain a marker that is involved in the processes in the body that we want to measure such as fat oxidation. The remaining will be excreted via urine and after measuring these they will be used to calculate the outcome. The stay in the respiration chamber is comfortable as it is designed as a hotel room and contains all devices to guarantee a pleasant stay. Subjects can leave the room whenever an emergency situation occurs. When two subjects participate at the same time there will not be a researcher present during the night, only in case of one subject the researcher will be present. Subjects are instructed on beforehand what to do in an emergency situation: where they have to walk to, which route they need to follow, which numbers to call and which alerts there are. Further security will be informed whenever there are subjects in the chambers.

Ethics

The protocol has to be approved by the Medical Ethical Committee of the University Hospital Maastricht/University Maastricht. The subjects will receive a written subject-information and will be informed orally on the study. A written informed consent will be obtained from all participants. They will be told that they can always stop without giving any reason. An independent medical doctor will be present for them to consult. The privacy of the subjects is guaranteed, using subject numbers throughout the study. All subject-characteristics will be encoded. Only the researcher and his supervisor have access to the code. Subjects can receive their personal results at the end of the study. The collected data will be saved for the time period that the

researcher needs to finish his PhD-project, this will be at least four years._After this four years the supervisor will store the data for an additional two years, probable end date will be 2013. Subjects will be insured via a subject insurance during their participation in the study and also for a period of four years after this study has been ended. A liability insurance has been closed as well.

For participation in this study subjects will receive financial compensation of 75 euro per visit, which is in total 150 euro, food during the stay in the respiration chamber and also their travel expenses will be compensated. Subjects are allowed to think for one week after they have received the information, before they give approval for participation. Within this research the Dutch law for medical-scientific research with humans (WMO) is of application.

Publication

The results will be published in the international peer reviewed literature. The CCMO Statement will be followed, which means that the results will be published within 3 months after analysis.

Study planning

Preparations	4 months
Measurements	6 months
Analyses	4 months
Report	2 months
Total duration	16 months

List of abbreviations

EE	-	Energy expenditure
GT	-	Green tea
EGCG	-	Epigallocatechingallate
SNS	-	Sympathetic nervous system
$COMT^{H/L}$	-	Catechol-O-methyl transeferase (high/ low)
cAMP	-	cyclic Amino mono phosphate
PKA	-	Phospho kinase A
PDE	-	Phosphodiesterase
Val	-	Valine
Met	-	Methionine
BMI	-	Body mass index
TFEQ	-	Three factor eating questionnaire
TEE	-	Total energy expenditure
ANOVA	-	Analysis of variance
SMR	-	Sleeping metabolic rate
DEE	-	Diet-induced energy expenditure
AEE	-	Activity-induced energy expenditure
RQ	-	Respiratory quotient
RMR	-	Resting metabolic rate
BMR	-	Basic metabolic rate
PAL	-	Physical activity level
O2	-	Oxygen
CO2	-	Carbon dioxide
H2SO4	-	Sulfuric acid
VAS	-	Visual analogue scale
AUC	-	Area under curve
WMO	-	Wet medisch-wetenschappelijk onderzoek
MEC	-	Medical ethical committee
CCMO	-	Centrale Commissie Mensgebonden Onderzoek

References

1. *Obesity: preventing and managing the global epidemic. Report of a WHO consultation.* World Health Organ Tech Rep Ser, 2000. **894**: p. i-xii, 1-253.
2. Stunkard, A.J., *Current views on obesity.* Am J Med, 1996. **100**(2): p. 230-6.
3. Wadden, T.A., A.J. Stunkard, and J. Liebschutz, *Three-year follow-up of the treatment of obesity by very low calorie diet, behavior therapy, and their combination.* J Consult Clin Psychol, 1988. **56**(6): p. 925-8.
4. Pasman, W.J., et al., *Effect of exercise training on long-term weight maintenance in weight-reduced men.* Metabolism, 1999. **48**(1): p. 15-21.
5. Graham, H.N., *Green tea composition, consumption, and polyphenol chemistry.* Prev Med, 1992. **21**(3): p. 334-50.
6. Weisburger, J.H., *Tea and health: a historical perspective.* Cancer Lett, 1997. **114**(1-2): p. 315-7.
7. Kao, Y.H., R.A. Hiipakka, and S. Liao, *Modulation of endocrine systems and food intake by green tea epigallocatechin gallate.* Endocrinology, 2000. **141**(3): p. 980-7.
8. Dulloo, A.G., J. Seydoux, and L. Girardier, *Potentiation of the thermogenic antiobesity effects of ephedrine by dietary methylxanthines: adenosine antagonism or phosphodiesterase inhibition?* Metabolism, 1992. **41**(11): p. 1233-41.
9. Dulloo, A.G., et al., *Normal caffeine consumption: influence on thermogenesis and daily energy expenditure in lean and postobese human volunteers.* Am J Clin Nutr, 1989. **49**(1): p. 44-50.
10. Astrup, A., et al., *Caffeine: a double-blind, placebo-controlled study of its thermogenic, metabolic, and cardiovascular effects in healthy volunteers.* Am J Clin Nutr, 1990. **51**(5): p. 759-67.
11. Bracco, D., et al., *Effects of caffeine on energy metabolism, heart rate, and methylxanthine metabolism in lean and obese women.* Am J Physiol, 1995. **269**(4 Pt 1): p. E671-8.
12. Chantre, P. and D. Lairon, *Recent findings of green tea extract AR25 (Exolise) and its activity for the treatment of obesity.* Phytomedicine, 2002. **9**(1): p. 3-8.
13. Dulloo, A.G., et al., *Efficacy of a green tea extract rich in catechin polyphenols and caffeine in increasing 24-h energy expenditure and fat oxidation in humans.* Am J Clin Nutr, 1999. **70**(6): p. 1040-5.
14. Dulloo, A.G., et al., *Green tea and thermogenesis: interactions between catechin-polyphenols, caffeine and sympathetic activity.* Int J Obes Relat Metab Disord, 2000. **24**(2): p. 252-8.
15. Diepvens, K., K.R. Westerterp, and M.S. Westerterp-Plantenga, *Obesity and thermogenesis related to the consumption of caffeine, ephedrine, capsaicin, and green tea.* Am J Physiol Regul Integr Comp Physiol, 2007. **292**(1): p. R77-85.
16. Hodgson, J.M., et al., *Is reversal of endothelial dysfunction by tea related to flavonoid metabolism?* Br J Nutr, 2006. **95**(1): p. 14-7.
17. Palmatier, M.A., A.M. Kang, and K.K. Kidd, *Global variation in the frequencies of functionally different catechol-O-methyltransferase alleles.* Biol Psychiatry, 1999. **46**(4): p. 557-67.

18. Kovacs, E.M., et al., *Effects of green tea on weight maintenance after body-weight loss.* Br J Nutr, 2004. **91**(3): p. 431-7.
19. Stunkard, A.J. and S. Messick, *The three-factor eating questionnaire to measure dietary restraint, disinhibition and hunger.* J Psychosom Res, 1985. **29**(1): p. 71-83.
20. Westerterp-Plantenga, M.S., et al., *Satiety related to 24 h diet-induced thermogenesis during high protein/carbohydrate vs high fat diets measured in a respiration chamber.* Eur J Clin Nutr, 1999. **53**(6): p. 495-502.
21. Schoffelen, P.F., et al., *A dual-respiration chamber system with automated calibration.* J Appl Physiol, 1997. **83**(6): p. 2064-72.
22. Brouwer, E., *On simple formulae for calculating the heat expenditure and the quantities of carbohydrate and fat oxidized in metabolism of men and animals, from gaseous exchange (Oxygen intake and carbonic acid output) and urine-N.* Acta Physiol Pharmacol Neerl, 1957. **6**: p. 795-802.
23. Westerterp, K.R., S.A. Wilson, and V. Rolland, *Diet induced thermogenesis measured over 24h in a respiration chamber: effect of diet composition.* Int J Obes Relat Metab Disord, 1999. **23**(3): p. 287-92.
24. Hursel, R. and M.S. Westerterp-Plantenga, *Green tea catechin plus caffeine supplementation to a high-protein diet has no additional effect on body weight maintenance after weight loss.* Am J Clin Nutr, 2009. **89**(3): p. 822-30.
25. Jian, L., A.H. Lee, and C.W. Binns, *Tea and lycopene protect against prostate cancer.* Asia Pac J Clin Nutr, 2007. **16 Suppl 1**: p. 453-7.
26. Smeets, A., M. Lejeune, and M. Westerterp-Plantenga, *The second meal effect of oral fat perception compared to fat ingestion on energy expenditure, hormones and appetite profile.* Submitted, 2009.
27. Westerterp, K.R., *Dietary fat oxidation as a function of body fat.* Curr Opin Lipidol, 2009. **20**(1): p. 45-9.
28. Westerterp, K.R., et al., *Dietary fat oxidation as a function of body fat.* Am J Clin Nutr, 2008. **87**(1): p. 132-5.
29. Votruba, S.B., S.M. Zeddun, and D.A. Schoeller, *Validation of deuterium labeled fatty acids for the measurement of dietary fat oxidation: a method for measuring fat-oxidation in free-living subjects.* Int J Obes Relat Metab Disord, 2001. **25**(8): p. 1240-5.
30. Harris, J.A. and F.G. Benedict, *A Biometric Study of Human Basal Metabolism.* Proc Natl Acad Sci U S A, 1918. **4**(12): p. 370-3.
31. Fahndrich, E. and M. Linden, *[Reliability and validity of the Visual Analogue Scale (VAS) (author's transl)].* Pharmacopsychiatria, 1982. **15**(3): p. 90-4.
32. Berube-Parent, S., et al., *Effects of encapsulated green tea and Guarana extracts containing a mixture of epigallocatechin-3-gallate and caffeine on 24 h energy expenditure and fat oxidation in men.* Br J Nutr, 2005. **94**(3): p. 432-6.
